# The Town Dweller

**Published:** 1890-03-15

**Authors:** 


### The Town Dweller.

*(Continued from page 340.)*

### LIFE IN TOWN AND COUNTRY.

The perusal of such a work as Dr. Fothergill's "Town Dweller," reviewed in our columns a fortnight ago, naturally suggests a comparison between town and country life. One cannot but feel that Dr. Fothergill himself would probably have disdained to institute such a comparison at all; and it is of course obvious enough that man, in his natural and primitive state, is a country animal, and that in the country alone he can attain full physical development. Still, it is equally obvious that civilisation has a tendency to bring men together in large and ever-increasing aggregations—in other words, in towns; and for believers in progress, it is impossible to hold, with some fanatics on the subject, that the tendency of mankind to congregate together is an unmixed evil.

But to return to our comparison. In the matter of air, the townsman is of course nowhere; his slight, stunted frame, and unhealthy, colourless complexion tell at once their melancholy tale, when placed beside the brawny countryman, whose cheeks are ruddy with the health borne to him on the wings of the pure fresh airs that frolic round his plough, or scamper in sudden gusts about his cottage, carrying away with them whatever is unhealthful or unclean. But pass from his air to his water supply, and you may find the countryman much worse off than his cousin in the town. The drinking water of a village is often conspicuous by its absence; and the badness, as well as the deficiency, of the water supply of many a country place is really a disgrace to the owners of property there. They who have met the village women toiling day after day, a pail in either hand, through the clayey fields (and only those who live on a clayey soil have any idea what "mud" really means) to a spring some third of a mile away, and appropriately called "The Dawdle"—they, we say, will hardly be disposed to think the country better off than the towns in this respect. As to house accommodation, while freely admitting that the country cottager is by far the better off of the two, we cannot pronounce his condition to be altogether a satisfactory one. His bedrooms are often little better than lofts, with no fireplace, and no door at the head of the staircase to keep out the draughts—not to mention the breakneck character of that staircase, which must, one would think, weed out a child or two now and again from the well be-olive-branched table. It must be remembered, too, that laid-on water and gas greatly increase the comfort, if not the healthiness, of a home. And it can hardly conduce to comfort to live—as many country people do—in cottages too old and tumble-down to bear repairing; so old, indeed, that the writer has found the tenant of one of a pair of lath-and-plaster cottages breaking off bits of the adjoining cottage—which had become altogether uninhabitable—and lighting her fire with them.

How to provide good cottage accommodation for the country labourer is a really difficult question; for we are told on all sides that, with the present rate of wages, the building of really good cottages cannot by any possibility pay; and although there may be good landlords here and there who will be content with one or two per cent. on the money laid out on cottage property, we can hardly expect that their good example will be largely followed. Facts seem to show, however, that it is the *small* owners of cottages who are most mean, and most careless of the decency and comfort of their tenants; so it is to be hoped that the landowners will not try to solve the problem by leaving it to others, difficult and unpleasant though it may be. Professor Buckman once suggested a novel and courageous mode of meeting the difficulty, in a paper read before the Dorset Chamber of Agriculture. Whether it would answer is a question on which we do not venture to give an opinion. He says:—

"My own notion is simply this: If I owned one of these parishes, as I find many gentlemen in Dorsetshire do, I should at once double the rent of every cottage. Whether I should get the money, I don't know; but I would double the rent, and I would ask my farmers to double the rent of every cottage they let to their labourers. I would take care I made the cottages doubly as good as they are at present. You would thus have better labourers, and I believe it would be worth your while—I know it would be worth my while—to add another shilling a-week to the labour list, with the idea that it should be paid with reference to improved cottages. This improvement would lower the rates very considerably; there would be less sickness, less illness, less unpleasant concomitants with reference to our parishes."

We pass on to the question of food. In one respect, the countryman is far more fortunate than the townsman—he can get a fresh and plentiful supply of vegetables daily from his own garden, and that is an advantage not lightly to be despised. But except for this, we doubt whether his food and drink are better or more wholesome than those of the town dweller. Where his wife bakes at home, he may be pretty sure of having pure bread; but he and his family live far too exclusively on bread, and it is a question whether baking at home does not foster this habit. The flour is got in by the sack; it is always at hand, and money seldom is; and so it comes to pass that the far more nourishing diet which might be procured is, partly from ignorance, but more from inertness and want of enterprise, never tried.

Tea, again, is even more drunk in the country than in the towns. It often goes with every meal; and when one sees the pot brewing for hours on the hob, one is almost glad to reflect on the slenderness of the purse which will not allow of this tea—this infusion of tannin, rather—being anything but weak. As to milk, many cottage children hardly know the taste of it; while of their fathers it may be said that they often know as little of the taste of wholesome, unadulterated beer, so successfully does the publican disguise it. Anybody who would be at the pains of seeking out means for securing to the country cottager a better diet (to a certain extent Parliament has done this already in the matter of beer), in which oatmeal, milk, and brown or whole-meal bread should certainly figure, would be doing a useful work.

But to turn from matters which chiefly affect the physique, to such as concern the moral and mental development of mankind. It is a truism to say that town life has a remarkably stimulating effect upon the brain, making the merest child a prodigy of sharpness, resourcefulness, and almost startling all-there-ness, as compared with even an adult country cousin. As for town tongues, they are so sharp that it is found advisable to recruit the metropolitan police from towns, the country bumpkin being often unable to resist the temptation of answering the cockney's running fire of chaff with his sturdy fist, instead of with his halting tongue. But while the town dweller is sharp, he cannot be said to be deep or reflective. Dr. Fothergill pronounces his joys and sorrows to be short-lived and on the surface, and says that the Cockney, at any rate, entirely lacks the spirituality which shows itself, feebly though it may be, in the countryman's attendance at religious services and evident enjoyment of hymns. And is not this just what we might expect? Man was made for two worlds—the world of nature and the world of men. Where he is entirely cut off from the former, how can we expect him to develop the finer and more reflective qualities of the mind—to feel that presence which shall "disturb him with the joy of elevated thoughts"? Even Wordsworth must have the remembrance of his "sylvan Wye" to be ever with him in the midst of his town life—to be to him

> "The anchor of my purest thoughts, the nurse,
> The guide, the guardian of my heart, and soul
> Of all my moral being."

How much more, then, must we, the "common herd," need such remembrances, to raise and purify our intercourse with our fellow men, and our own personal life? And although the taciturnity and unresponsiveness of the country labourer make it hard to discover what effect the "lonely hills," the woods and rivers, really have upon him, we can hardly doubt that, all unconsciously though it may be, he does drink in some of their beauty, and mirror it, if ever so dimly, in his thoughts and feelings. At least, he is free from the sounds and sights of wickedness with which the townsman must needs be familiar. In a world where, as Bishop Heber has it, "every prospect pleases, and only man is vile," he has all the brightness and the beauty, and need know next to nothing of man, except through himself. Then, again, he has solitude—that solitude which has been the very life of such thinkers as the author of *Thorndale*, and without which it is difficult to conceive that any human nature can develop its best and finest qualities. We do not mean, of course, that a life of solitude is necessary or even advisable for most men; but those who are used to a life spent, partially at least, in the country, cannot but start and shudder when it suddenly comes home to them that the great mass of townspeople live for ever in the midst of a noisy, bustling throng, with never a minute in the day or night when they can breathe the wide air, stretch their arms into open space, and feel themselves alone with Nature and with God. True, they may have their corners rubbed off, their wits sharpened, by constant contact with their neighbours; but that is a long price to pay for the solitude and peace which make the countryman so unsophisticated, so child-like, but which the townsman would not know how to enjoy were it offered to him.

It is difficult to say how far the countryman's superior morality springs from real virtue, or from a mere ignorance of the various dodges which townsmen have invented for doing wrong, and then escaping the consequences of that wrong-doing. We do think, however, that a countryman is not only less crafty, but less set on securing every advantage for himself at the expense of others; and that his domestic affections are stronger. Those who have seen the simple delight with which a country labourer will take his youngest-born—the seventh or eighth, it may be—into his arms, not even the presence of a visitor hindering him from bestowing many a furtive kiss on it in the intervals of munching the hunk of bread which, with a bit of butter or dripping, forms his dinner—those who have seen this, we say, cannot but think him more affectionate, as well as more simple, than the average townsman.

On the other hand, the slowness of brain and general lumpishness of those who always live in the country is certainly a drawback. Even educated people cannot dispense with the general rub-up which an occasional visit to town friends will give them. The writer has more than once heard it remarked by persons who lived a quiet country life that their brains and tongues seemed to get rusty from want of use, and that for the first two or three days of intercourse with townsfolk they could but listen dumbly, as they were used to do with the books which made up all their intellectual pabulum at home.

So that what we come to is this: Town and country life both have their drawbacks, some of which, being removable, we should promptly seek to remove. As to those which are not, we can but keep in our minds Dr. Fothergill's remark that "our aristocracy betray no signs of physical degeneracy, because the greater portion of their childhood is spent in the country, and in mature age their visits to the country are numerous and prolonged;" and do all we can, both for ourselves and for the masses, to obliterate the hard-and-fast line which now separates town and country dwellers, and gives to each but a partially-developed life.

